# Supplementary material for: Probabilistic metaplasticity for continual learning with memristors in spiking networks
Source: Sci Rep. 2024 Nov 27;14:29496. doi: 10.1038/s41598-024-78290-w (PMC11603065; doi:10.1038/s41598-024-78290-w)
Supplement: Supplementary file 1 — Supplementary Information. [file 41598_2024_78290_MOESM1_ESM.pdf]

Supplementary Material

Supplementary note S1: Memory overhead computation

Continual learning models typically require additional parameters to alleviate catastrophic forgetting. We determine the memory overhead for parameters other than the network weights for different continual learning models[1,2]. Supplementary table S1 lists the details of memory overhead computation. The "Network Configuration" column shows the number of neurons per layer. "Network Type" and "Weight Precision" columns list the type of network and precision of weights, respectively. "MLP" stands for multi-layer perception and "SNN" denotes spiking neural network. The "CL Parameter Overhead" column lists the cumulative bit precision of the parameters needed in addition to the weights. These parameters may be required for every weight or neuron, which is denoted in parentheses by W and N, respectively. "Normalized Memory Overhead" column lists the memory overhead for the models considering a network with 784 input neurons, 200 hidden neurons and 2 output neurons. "Total Memory Overhead" column lists the memory overhead of the network used to evaluate the model, as listed in the "Network Configuration" column. For models with varying memory overhead, we consider the maximum limit.

Table S1. Memory overhead computation for different continual learning models.

Model	Network Configuration	Network Type	Weight Precision	CL Parameter Overhead	Normalized Memory Overhead	Total Memory Overhead
LwF[3]	784 - 200 - 200 - 2	MLP	32-bit	32-bit (W)	628.8 kB	788 kB
MAS[4]	784 - 200 - 200 - 2	MLP	32-bit	64-bit (W)	$\sim$ 1.2 MB	1.6 MB
BGD[5]	784 - 200 - 200 - 2	MLP	32-bit	64-bit (W)	$\sim$ 1.2 MB	1.6 MB
SS[6]	784 - 200 - 200 - 2	MLP	32-bit	32-bit (W)	628.8 kB	788 kB
TACOS[1]	784 - 200 - 200 - 2	SNN	32-bit	48-bit (W)	$\sim$ 0.9 MB	$1.2MB$
NEO[2]	784 - 400 - 400 - 2	MLP	32-bit	16-bit (W)	$\sim$ 0.3 MB	$\sim 1MB$
NAI[7]	784 - 400 - 400 - 2	MLP	32-bit	32-bit (N)	$\sim$ 0.8 kB	3.2 kB
Activity-dependent Metaplasticity w/ Gradient Accumulation	784 - 200 - 2	SNN	Multi-memristor weights	48-bit (W)	$\sim$ 0.9 MB	$\sim$ 0.9 MB
Probabilistic Metaplasticity	784 - 200 - 2	SNN	Multi-memristor weights	16-bit (W)<br>16-bit (N)	$\sim$ 0.3 MB<br>$\sim$ 0.4 kB	$\sim$ 0.3 MB<br>$\sim$ 0.4 kB

Table S2. Performance of a spiking network on the split-Fashion MNIST task in different settings. The table shows the individual task accuracies and the mean accuracy across tasks after sequential training. The mean and standard deviation were determined over 5 runs.

Task ID	Baseline	Random Consolidation	Decaying Probabilistic Plasticity			Probabilistic Metaplasticity
			(Factor=2)	(Factor=5)	(Factor=10)	
Task 1	49.94 ± 0.06	52.84 ± 2.74	50.13 ± 0.19	89.28 ± 2.80	94.07 ± 0.71	95.65 ± 0.24
Task 2	50.06 ± 0.15	50.21 ± 0.13	50.12 ± 0.22	57.74 ± 1.30	85.51 ± 2.04	79.02 ± 1.09
Task 3	88.22 ± 5.00	96.08 ± 0.67	97.75 ± 0.53	98.03 ± 0.58	87.44 ± 3.11	95.99 ± 1.42
Task 4	86.86 ± 9.84	98.69 ± 0.95	99.43 ± 0.30	99.92 ± 0.05	97.76 ± 0.67	98.77 ± 0.11
Task 5	99.67 ± 0.05	99.61 ± 0.07	99.38 ± 0.06	91.75 ± 1.32	72.61 ± 5.80	96.72 ± 0.35
Mean	74.95 ± 2.87	79.48 ± 0.76	79.36 ± 0.24	87.34 ± 0.88	87.48 ± 1.61	93.23 ± 0.14

Supplementary note S2: Controlled experiments on the split-Fashion MNIST task

Supplementary table S2 shows the results of the controlled experiments on the split-Fashion MNIST task. In random consolidation, the update probabilities of the weights are calculated as probabilistic metaplasticity, but the results are randomly shuffled among the weights. In decaying probabilistic plasticity, all weights are updated with the same probability and the update probability is reduced by a factor with each incoming task to help retain previous knowledge. All experiments were carried out considering a spiking network with a single hidden layer of 200 neurons and 7 memristor weights. The baseline network is trained with eRBP combined with error threshold. From Table S2, we see that random consolidation shows performance similar to the baseline with catastrophic forgetting of the initial two tasks. Decaying probabilistic plasticity either remembers old tasks well or learns new tasks well, depending on the decaying plasticity factor with a poor balance of plasticity and stability. Probabilistic metaplasticity shows

Supplementary Algorithm 1: Activity-dependent metaplasticity with gradient accumulation for continual learning. $\mathcal{T}$ denotes a set of sequential tasks. S^{in} and S^{out} are the input and output spike trains of the network and W denotes the memristor weights. U is the error accumulated at the dendritic compartments. As the network trains, the gradients of the weights eligible for update are accumulated in high-precision memory U_{syn}. When the accumulated gradient crosses a threshold U_{th}, the memristor weights are programmed to the next higher or lower conductance level depending on the sign of the accumulated error. The function Program() refers to the operations required to update memristor conductance based on the error.

```
▷
for task ∈ T do
    for {x^t, y^t} ∈ {X^t, Y^t} do
        for t ∈ T_sim do
            Forward Pass: S^out(t) ← f(S^in(t), W(t)
            Random Error Feedback: τ_U ∂U/∂t = −U + E R_U
            Update Neuron Trace: d/dt X_tr = −X_tr/τ_tr + S
            for i ∈ {X_i(t) == 1} do
                if I_min < I_j < I_max then
                    U_syn,ij ← −η U_j f(m_ij, w_ij)
                end
            end
            if |U_syn,ij| > U_th then
                Update memristor weights: W ← Write(R_mem, U)
                U_syn,ij = 0
            end
        end
        Update metaplasticity coefficient: m ← m + Δm
    end
end
```

superior balance as it remembers initial tasks while being able to learn the final task with lower accuracy degradation compared to decaying probabilistic plasticity.

Supplementary note S3: Energy analysis

Supplementary table S3 lists the design parameters for the energy analysis in the mixed-signal design. We designed a 16×16 crossbar with programming peripherals in the 65nm technology node with 1.2 V and 3.3 V supply voltage. The row and column peripherals consist of transmission gate-based multiplexers and level shifters to supply the appropriate voltages to the crossbar terminals required during different network operations. The memristor weights are implemented with a Verilog-A model that captures the behavior of the 1T1R memristor device considered in this work[8]. Operational amplifiers and voltage comparators were used for the read operation and boxcar function[9]. The modules needed for the operations carried out in the digital domain were synthesized in the IBM 65nm technology node considering 1.2 V supply and 100 MHz frequency in Synopsys Design Compiler.

Probabilistic Metaplasticity Approach: When training with probabilistic metaplasticity, the accumulated error is compared with the error threshold at each timestep during the spike train presentation. This operation was carried out with 16-bit digital comparator. If the error is above the threshold, the currents at the post-synpatic neurons are converted to voltage with an operational amplifier, which is then propagated to voltage comparators to evaluate the boxcar function. The energy for this operation is estimated considering the average number of active spikes in the network and assuming the memristor resistance to be the average of the resistance levels used to map weights. We consider a read voltage of 0.1 V with 100 ns duration. The average power consumption of the operational amplifier is $\sim 24\ \mu$W, with 1.2 V supply and $\sim 20\ \mu$A average current. The reference for the operational amplifier consumes $\sim 13.5\ \mu$W average power. The output of the operational amplifier is connected to the boxcar function unit, which consists of two voltage comparators and an AND gate. The energy is estimated as -

$$E_{\text{cc}} = \left(\frac{V_{\text{read}}^2}{R_{\text{weight,avg}}} + 2 \times P_{\text{opamp}} + P_{\text{reference}} + P_{\text{boxcar unit}}\right) \times t_{\text{read}}$$

Table S3. Mixed-signal design parameters for energy analysis.

Parameter	Value
Technology node	65 nm
Supply voltage	1.2 V, 3.3 V
Frequency	100 MHz
Memristor devices per weight	7
Memristor resistance range	3.5 $k\Omega$ - 25 $k\Omega$
Set/Reset/Read voltage	2/1.6/0.1 V
ADC resolution	8-bit

Table S4. Energy for network operations.

Operation	Energy Consumption (pJ)
Memristor weight update	38.6
Memristor weight read	23.8
Addition (16-bit/32-bit)	0.26/0.6
Multiplication (16, 8 / 16, 16/ 16, 32)	0.98/1.98/4
Shift operation	0.2
Comparison (16-bit / 32-bit)	0.24/0.48
Random number generation	0.42
Exponential metaplasticity function	7.962
0.6 MB SRAM 32-bit read/write	144.3/142.5
0.3 MB SRAM 16-bit read/write	124.1/103.7
39.4 kB SRAM 16-bit read/write	68.76/37.25
0.5 kB SRAM 16-bit read/write	5.4/6.3
Register access	0.04795

For the weights eligible for update, the update probability is computed and compared with a random number. If the random number is lower than the update probability of a memristor, it is updated. We designed a 32-bit Linear Feedback Shift Register (LFSR) to generate the random number. Since the update probability depends on the weight magnitude, this requires a read weight operation. During this operation, the columns of the crossbar are connected to an operational amplifier, the output of which can be read by an Analog to Digital Converter (ADC). We estimate the energy consumed by the memristor device assuming it to be the average of resistance levels used to map the weight. The energy for 8-bit ADC in 65nm technology node is estimated according to[10] to be 19.46 pJ. This leads to read weight energy determined as -

$$E_{\text{read weight}} = (P_{\text{opamp}} + P_{\text{reference}} + \frac{V_{\text{read}}^2 \times n_{\text{mem}}}{R_{\text{mem,avg}}}) \times t_{\text{read}} + E_{\text{ADC}}$$

The metaplasticity coefficients require 16-bit precision and are emulated with on-chip SRAM. The energy required to compute the exponential metaplasticity function was determined with a pipelined exponential function module[11]. The output of the function is compared with the random number generated with LFSR to determine which weights need to be updated.

The weight update involves programming the 1T1R devices, which can be set to different conductance levels by controlling the compliance current with the gate voltage in the transistor[12]. Each weight update operation consists of a read operation to determine the current conductance level, then a reset and set operation to program it to the next higher or lower conductance level based on the sign of the error. The read energy is estimated with the same considerations as the read weight operation. During set (reset), the selected row and column are set to 2 V (0 V) and 0 V (1.6 V), respectively, for 100 ns[13]. The set (reset) energy varies with the target (current) conductance level of the device. We determine the energy consumption for all conductance levels and use the average energy value for the analysis.

$$E_{\text{read}} = (P_{\text{opamp}} + P_{\text{reference}} + \frac{V_{\text{read}}^2}{R_{\text{mem,avg}}}) \times t_{\text{read}} + E_{\text{ADC}}$$

$E_{\text{set}} = P_{\text{set,avg}} \times t_{\text{set}}$
$E_{\text{reset}} = P_{\text{reset,avg}} \times t_{\text{reset}}$
$E_{\text{weight program}} = E_{\text{read}} + E_{\text{set}} + E_{\text{reset}}$

The metaplasticity coefficients are emulated as on-chip SRAM with 16-bit bus width. We determine the SRAM read and write energy with HP Cacti[14]. For individual, module-shared and neuron-shared metaplasticity coefficients, the on-chip SRAM blocks were instantiated as 0.3 MB, 39.4 kB and 0.5 kB respectively. The layer-shared metaplasticity coefficients were realized with registers.

Activity-dependent metaplasticity with gradient accumulation: The gradient accumulation approach computes the gradients of the weights eligible for update according to eRBP. Checking the update eligibility requires evaluating the boxcar function and computing the gradient requires evaluating the exponential metaplasticity function. The energy for both these operations are determined in the same manner as the probabilistic approach. The output of the metaplasticity function (32-bit) is multiplied by the dendritic compartment error (16-bit), and the learning rate is modeled with a diadic number which replaces the multiplication with a shift operation. The calculated gradient is compared with the threshold to determine the weights to be updated and then accumulated in 32-bit on-chip SRAM. The on-chip SRAM required for the gradients and the metaplasticity coefficients were modeled as 0.6 MB block with 32-bit bus width and 0.3 MB block with 16-bit bus width, respectively.

The energy estimates for the different operations for the analysis are listed in Supplementary table S4.

Table S5. Performance of a spiking network with probabilistic metaplasticity with individual and shared metaplasticity coefficients on the split-MNIST task. The table shows the mean and standard deviation of the individual task accuracies and the mean accuracy across tasks over 5 runs after sequential training.

#Task	Individual Metaplasticity Coefficients	Module-shared Metaplasticity Coefficients	Neuron-shared Metaplasticity Coefficients	Layer-shared Metaplasticity Coefficients
Task 1	84.95 ± 3.42	88.77 ± 4.21	90.00 ± 3.65	89.49 ± 7.37
Task 2	90.20 ± 1.96	86.71 ± 4.14	90.04 ± 2.33	88.49 ± 5.78
Task 3	72.06 ± 2.60	68.67 ± 3.99	70.11 ± 4.27	69.16 ± 5.83
Task 4	94.73 ± 0.94	93.21 ± 0.74	91.53 ± 2.99	89.06 ± 1.39
Task 5	76.54 ± 2.60	70.16 ± 4.77	68.68 ± 4.64	62.49 ± 3.34
Mean	83.70 ± 0.78	81.50 ± 1.18	82.07 ± 0.84	79.74 ± 1.21

Table S6. Classification accuracies of a spiking network with multi-memristor weights ($n_{mem} = 7$) on the split-MNIST and split-Fashion MNIST tasks.

	Split-MNIST		Split-Fashion MNIST	
# Task	Gradient Accumulation	Error-threshold training	Gradient Accumulation	Error-threshold training
Task 1	99.91 ± 0.07	99.93 ± 0.02	97.43 ± 0.66	96.35 ± 1.58
Task 2	96.52 ± 0.86	96.56 ± 0.91	96.07 ± 0.95	96.28 ± 0.51
Task 3	98.22 ± 0.45	98.93 ± 0.18	99.84 ± 0.12	99.94 ± 0.04
Task 4	99.58 ± 0.14	99.51 ± 0.09	99.93 ± 0.04	99.97 ± 0.02
Task 5	96.31 ± 1.01	97.57 ± 0.31	99.61 ± 0.18	99.69 ± 0.04

References

1. Soures, N., Helfer, P., Daram, A., Pandit, T. & Kudithipudi, D. TACOS: Task Agnostic Continual Learning in Spiking Neural Network. In *Theory and Foundation of Continual Learning Workshop at ICML'2021* (July 2021).
2. Daram, A. & Kudithipudi, D. NEO: Neuron State Dependent Mechanisms for Efficient Continual Learning. In *Proceedings of the 2023 Annual Neuro-Inspired Computational Elements Conference*, NICE '23, 11–19, DOI: 10.1145/3584954.3584960 (Association for Computing Machinery, New York, NY, USA, 2023).
3. Li, Z. & Hoiem, D. Learning without Forgetting. *IEEE Transactions on Pattern Analysis Mach. Intell.* **40**, 2935–2947, DOI: 10.1109/TPAMI.2017.2773081 (2018).

4. Aljundi, R., Babiloni, F., Elhoseiny, M., Rohrbach, M. & Tuytelaars, T. Memory Aware Synapses: Learning what (not) to forget. In *Proceedings of the European Conference on Computer Vision (ECCV)* (2018).

5. Zeno, C., Golan, I., Hoffer, E. & Soudry, D. Task Agnostic Continual Learning Using Online Variational Bayes. *arXiv preprint arXiv:1803.10123* (2018).

6. Schug, S., Benzing, F. & Steger, A. Presynaptic stochasticity improves energy efficiency and helps alleviate the stability-plasticity dilemma. *eLife* **10**, e69884, DOI: 10.7554/eLife.69884 (2021).

7. Kim, S. & Lee, S. Continual Learning with Neuron Activation Importance. In Sclaroff, S., Distante, C., Leo, M., Farinella, G. M. & Tombari, F. (eds.) *Image Analysis and Processing – ICIAP 2022*, 310–321 (Springer International Publishing, Cham, 2022).

8. Amer, S., Sayyaparaju, S., Rose, G. S., Beckmann, K. & Cady, N. C. A practical hafnium-oxide memristor model suitable for circuit design and simulation. In *2017 IEEE International Symposium on Circuits and Systems (ISCAS)*, 1–4, DOI: 10.1109/ISCAS.2017.8050790 (2017).

9. Zyarah, A. M. & Kudithipudi, D. Neuromemristive multi-layer random projection network with on-device learning. In *2019 International Joint Conference on Neural Networks (IJCNN)*, 1–8, DOI: 10.1109/IJCNN.2019.8851735 (2019).

10. Tripathi, V. & Murmann, B. An 8-bit 450-ms/s single-bit/cycle sar adc in 65-nm cmos. In *2013 Proceedings of the ESSCIRC (ESSCIRC)*, 117–120, DOI: 10.1109/ESSCIRC.2013.6649086 (2013).

11. Hussain, M. A., Lin, S.-W. & Tsai, T.-H. An area-efficient and high throughput hardware implementation of exponent function. In *2022 IEEE International Symposium on Circuits and Systems (ISCAS)*, 3369–3372, DOI: 10.1109/ISCAS48785.2022.9937238 (2022).

12. Liehr, M., Hazra, J., Beckmann, K., Rafiq, S. & Cady, N. Impact of Switching Variability of 65nm CMOS Integrated Hafnium Dioxide-based ReRAM Devices on Distinct Level Operations. In *2020 IEEE International Integrated Reliability Workshop (IIRW)*, 1–4 (IEEE, 2020).

13. Hazra, J. *et al.* Optimization of switching metrics for cmos integrated hfo2 based rram devices on 300 mm wafer platform. In *2021 IEEE International Memory Workshop (IMW)*, 1–4, DOI: 10.1109/IMW51353.2021.9439618 (2021).

14. Muralimanohar, N., Balasubramonian, R. & Jouppi, N. P. Cacti 6.0: A tool to model large caches. *HP laboratories* **27**, 28 (2009).
